# Predicting High Urinary Tract Infection Rates in Skilled Nursing Facilities: A Machine Learning Approach

**DOI:** 10.3390/healthcare13202632

**Published:** 2025-10-20

**Authors:** Diane Dolezel, Tiankai Wang, Denise Gobert

**Affiliations:** 1 Health Informatics & Information Management Department, Texas State University, Round Rock, TX 78665, USA; tw26@txstate.edu; 2 Physical Therapy Department, Texas State University, Round Rock, TX 78665, USA; dgobert@txstate.edu

**Keywords:** urinary tract infections, skilled nursing facilities, machine learning, predicting

## Abstract

**Objectives**: Urinary tract infections (UTIs) are the most common healthcare-associated infections in Skilled Nursing Facilities (SNFs); they are associated with longer lengths of stay, higher levels of care, increased treatment costs, and higher mortality rates. This study aimed to develop a machine learning classification model to predict the risk of high catheter-associated urinary tract infection rates based on SNF characteristics. **Methods**: We analyzed 94,877 total SNF-year observations from 2019 to 2024, not unique facilities; thus, individual SNFs may appear in multiple years. The factor variables were average length of stay in days, number of staffed beds, total nurse and total physical therapy staffing hours per resident per day, facility ownership, geographic classification, facility accreditation, Accountable Care Organization affiliations, Centers for Medicare and Medicaid Services SNF Overall Star Rating, and the SNF-year of the observations. We utilized three machine learning models for this analysis: Random Forest, XGBoost, and LightGBM. We used Shapley Additive exPlanations to interpret the best-performing machine learning model by visualizing feature importance and examining the relationship between key predictors and the outcome. **Results**: We found that machine learning models outperformed traditional logistic regression in predicting UTIs in skilled nursing facilities. Using the best-performing model, Random Forest, we identified rural SNFs, and the number of staffed beds as the most influential predictors of high UTI rates, followed by average length of stay, and geographic location. **Conclusions**: This study demonstrates the value of using facility-level characteristics to predict the risk of UTIs in SNFs with machine learning models. Results from this study can inform infection prevention efforts in post-acute care settings.

## 1. Introduction

Skilled nursing facility (SNF) residents are vulnerable to urinary tract infections (UTIs) due to advanced age, increased use of indwelling catheters, cognitive deficits, and limited mobility [1,2]. UTIs are the most common healthcare-associated infection (HAI) in SNFs; they are associated with longer lengths of stay, higher levels of care, increased treatment costs, and higher mortality rates [3,4]. Prediction of UTIs in SNFs could improve patient outcomes, reduce resource waste, and treatment costs, but relevant evidence-based predictive machine learning studies are scarce [5]. A few machine learning (ML) studies considered UTI rates in intensive care units [6,7,8] and hospitals [9] based on patient clinical and sociodemographic characteristics. Other studies associated HAIs with hospital occupancy, hospital type, nurse staffing, and hospital ownership [5,10]. What is not well understood are the SNF characteristics associated with a high risk of healthcare-associated infections.

This study aims to enhance our understanding of the risk factors for catheter-associated UTIs based on skilled nursing facility (SNF) characteristics using machine learning modeling. The findings will inform healthcare managers seeking to proactively reduce UTIs through targeted interventions, as well as practitioners responsible for implementing these strategies. Thus, to guide this study, we hypothesize the following:

1. A machine learning model incorporating SNF characteristics will outperform traditional logistic regression in predicting facilities at high risk for HAIs.
2. Specific facility-level characteristics, such as geographic location, number of staffed beds, and average length of stay, are significant predictors of urinary tract infections in skilled nursing facilities.

### 1.1. Background

HAIs are acquired during treatment and pose a serious threat to patient safety and healthcare outcomes. They represent a significant public health issue and contribute billions of dollars annually to the operational costs of U.S. healthcare systems [11]. HAIs prolong the length of stay, necessitating more intensive treatment and additional diagnostic testing, and often result in unreimbursed costs for healthcare [12]. Reducing HAI rates is a top priority for the Centers for Disease Control and Prevention and its healthcare partners, as outlined in the National Action Plan to Prevent Healthcare-Associated Infections [13,14,15].

HAIs are costly to treat due to their high incidence among debilitated patients, like SNF patients, those in intensive care units, and individuals with multiple comorbidities [16]. HAIs are challenging to treat because many are caused by multidrug-resistant organisms. Additionally, sepsis, a life-threatening condition, commonly occurs in patients affected by these infections [17]. High HAI rates carry financial penalties. Under the Centers for Medicare & Medicaid Services (CMS) Hospital-Acquired Condition Reduction Program, Medicare payments are reduced based on a hospital’s performance on hospital-acquired condition metrics [12]. Measures tracked by this program include Central Line-Associated Bloodstream Infection, Catheter-Associated Urinary Tract Infection, Colon and Abdominal Hysterectomy Surgical Site Infection (SSI), Methicillin-resistant *Staphylococcus aureus* bacteremia, and *Clostridium difficile* Infection [12].

There is concern that HAIs will be more prevalent in SNFS, as the risk of infection increases with age, and the U.S. elderly population is projected to double in 2025 as compared to 2012 [18]. Due to the elderly population’s increased acuity, weakened immunity, polypharmacy, and the ongoing nursing shortage, rising HAI rates are expected to strain clinical care and SNF finances.

### 1.2. Gap in the Literature

Machine learning is a subset of artificial intelligence focused on algorithmic analysis; it has been useful in improving predictions in healthcare outcomes [19,20,21,22]. Most prior studies used traditional logistic regression to predict the risk of HAI [23]. In recent years, the landscape of predictive modeling has been notably reshaped by advancements in machine learning. Researchers have found evidence that machine-learning models outperform logistic regression in outcome prediction [24,25]. Several studies have used machine learning to identify the risks of a particular HAI, such as surgical site infections [26] or hospital-acquired urinary tract infection [9] or healthcare-associated pneumonia [27] based on patient-related characteristics (e.g., age, gender, comorbidities), hospital units (e.g., Intensive Care Units), and treatments (length of stay, procedures, antibiotic use). One study examined a few hospital characteristics like patient safety, climate, standard precaution adherence, level and type of nurse staffing, and hospital ownership on HAI levels [10].

Despite ML models being used for HAIs, most studies have explored UTIs in hospitals or nursing homes. SNF facility characteristics associated with higher rates of UTIs are understudied. A few studies have explored how SNF factors, such as patient safety, climate adherence to standard precautions, nurse staffing, and ownership, impact their HAI rates [10]. This research extends work on UTIs to SNFs [3,28].

### 1.3. Aim of the Study

The primary objective of this study is to develop a machine learning model to predict skilled nursing facility characteristics associated with high rates of catheter-associated urinary tract infections. The study aims to identify the most effective machine learning model that outperforms traditional logistic regression in predicting SNFs at high risk for high UTI rates based on facility characteristics. A secondary objective is to determine the most influential factors in predicting SNFs with a high risk of UTIs.

## 2. Materials and Methods

### 2.1. Study Design and Data Collection

This predictive study employed a machine learning approach to develop a classification model that distinguishes skilled nursing facilities (SNFs) with high UTI rates from those with low rates, based on facility-level characteristics.

Data were sourced from the university’s licensed access to the Definitive Healthcare dataset (2019–2024), which consolidates information from publicly accessible databases on SNFs across the United States. These sources include: American Hospital Association Annual Survey (hospital characteristics), Medicare Cost Report (financial metrics), and the Hospital Value-Based Purchasing Program (quality indicators) [29].

From this dataset, we extracted 94,877 SNF-year observations from 2019 to 2024. The dataset includes repeated yearly entries for the SNFs, with 14,166, unique SNFs, indicating that many facilities contributed data across multiple years.

Definitive Healthcare reports the percentage of UTIs for each facility. For classification, a binary outcome variable was created as “high_UTI” (coded as 1) if the UTI rate exceeded the 75% quartile in the training dataset (2019–2023), and “low_UTI” (coded as 0) otherwise. The primary objective was to identify the optimal machine learning model for predicting SNF characteristics associated with elevated UTI risks.

### 2.2. Factor Variables

Facility-level factors were selected for their clinical relevance and data availability. These predictors included

- alos: Average length of stay in days (continuous);
- num_staffed_beds: Number of staffed beds (continuous);
- nurse_hrs: Total nurse staffing hours per resident per day (continuous);
- pt_hrs: Physical therapist (PT) staffing hours per resident per day (continuous);
- ownership: facility ownership type, categorical (values included: ‘Proprietary-Partnership’, ‘Proprietary-Other’, ‘Proprietary-Individual’, ‘Proprietary-Corporation’, ‘Voluntary Nonprofit-Other’: 0, ‘Voluntary Nonprofit-Church’, ‘Governmental-Hospital District’, ‘Governmental-County’: 0, ‘Governmental-State’, ‘Governmental-Other’, ‘Governmental-Federal’, ‘Governmental-City’, ‘Governmental-City-County’;
- geographic classification: Rural or urban classification (categorical: rural = 1 or urban = 0);
- accreditation: facility accreditation agency, if any (binary: accredited = 1, not accredited = 0);
- aco_affiliations: Accountable Care Organization (ACO) affiliation (binary: affiliated = 1, not affiliated = 0);
- star_rating: CMS SNF Overall Star Rating (categorical: 1 to 5);
- year: the SNF-year of observations (categorical ordinal: 2019, 2020, 2021, 2022, 2023, 2024).

These variables reflect operational, organizational, and quality-related characteristics that the study researchers consider relevant predictors of UTI risk. For example, larger hospitals (measured by bed count) can have higher infection rates due to larger case volumes [30]. Longer than average hospital stays can increase exposure to infectious agents, resulting in a higher risk of HAIs. Nurse staffing shortages, especially in rural hospitals, have been associated with higher HAI rates [31]. Hospital ownership type has been explored in relation to adverse events, with for-profit and government-owned hospitals reporting higher rates of adverse events compared to nonprofit hospitals [32]. Accountable Care Organizations are noted for their evidence-based care coordination, such as assigning case managers to high-risk patients, which may lower HAI rates [33]. CMS Star Ratings have been associated with infection control procedures [34].

The physical therapy hours per resident day are associated with UTI risk because increased mobility, even within the bed or from bed to chair, can reduce UTI risk in residents. Improved mobility supports better bladder emptying and hygiene, potentially reducing UTI risk. Conversely, reduced mobility, sometimes a result of certain physical therapy interventions, may increase UTI risk if it impairs bladder emptying or leads to hygiene challenges. Evidence indicates that maintaining or enhancing mobility (such as walking, repositioning, or in-bed movements) can reduce UTI risk by up to 69% during hospitalization [35]. The same study also noted a 38% to 80% reduction in UTI risk among SNF residents with severe mobility impairments, such as those who are wheelchair-bound or have limb amputations, when they received physical therapy. Additionally, emerging research supports the role of specialized physical therapy, such as pelvic floor therapy, in addressing voiding dysfunction and recurrent UTIs, providing a targeted intervention for managing these conditions [36].

### 2.3. Data Collection and Preprocessing

#### 2.3.1. Categorical Encoding

To prepare the dataset for machine learning, categorical variables were recoded for consistency and interpretability. The year was encoded as an ordinal categorical feature. The CMS Star Rating was treated as an ordinal categorical variable, ranked 1–5. Geographic_classification was binarized, with urban coded as 1, and rural as 0.

Facility ownership was recoded into two binary variables according to CMS definitions: for-profit ownership (1 = yes, 0 = no), where proprietary facilities are classified as privately owned, for-profit entities, and government_hospital (1 = yes, 0 = no), indicating a facility is publicly owned and operated by a government entity.

#### 2.3.2. Missingness Analysis and Imputation

Table 1 presents the extent of missingness by variable and year with the imputation strategy after recoding. The following variables had no missing data and were not imputed: geographic classification, UTI rate, and year.

Variables with low missingness (0–5%) were handled with simple imputation. Numeric variables (alos, num_staffed_beds) were imputed with the median, while the categorical variables (for_profit, government_owned, star_rating) were imputed with the mode. Variables with high missingness (>90%) were excluded from the analysis. These included: accreditation, aco_affiliations, nurse_hrs, and pt_hrs.

### 2.4. Outlier Detection and Handling

To reduce skewness and diminish the influence of extreme values, all continuous numeric features were assessed for outliers using the interquartile range (IQR) method. Observations falling below the first quartile minus 1.5 times the IQR or above the third quartile plus 1.5 times the IQR were flagged as mild outliers. Winsorization was applied to cap these values at their respective lower and upper bounds, thereby retaining all observations while minimizing the impact of outliers on model training.

The following variables were Winsorized, with the number of outliers capped noted in parentheses: alos (7134), alos_imputed (7717), and number of staffed beds (4714) and num_staffed_beds_imputed (5171)

This approach ensured that the dataset remained robust and suitable for predictive modeling without distortion from extreme values.

### 2.5. Multicollinearity and Target Encoding

All features were examined for multicollinearity using the variance inflation factor (VIF) analysis. The maximum VIF observed was 1.48, well below the commonly accepted threshold of 5, indicating no substantive multicollinearity concerns, as shown in Table 2.

We defined high_UTI as the top quartile of the UTI rate among observations from 2019 to 2023. This criterion was applied consistently to both the training data (2019–2023) and the held-out test data (2024). We constructed a binary target: UTI_high for observations in the top 25% of the distribution, and UTI_low for the remaining 75%. This quartile-based definition yields an expected prevalence of approximately 25%, resulting in only a modest class imbalance that is readily manageable in standard classification analyses. To address this, we applied safeguards such as stratified data partitioning and imbalance-aware evaluation metrics.

Model assessment emphasized precision, recall, F1 score, PR AUC, and balanced accuracy. We did not adopt a decile-based definition (top 10%), as this would reduce the positive-class prevalence to roughly 10%, creating a strongly imbalanced 1:9 ratio. Such an imbalance could compromise model stability and interpretability without resorting to more aggressive corrective techniques.

### 2.6. Data Analysis

Descriptive statistics were calculated using frequencies and percentages. In this study, we employed multiple predictive modeling approaches to identify factors associated with high urinary tract infection (UTI) rates in skilled nursing facilities. Logistic regression was used as the baseline model due to its interpretability and widespread use in clinical research. In addition to this traditional method, we implemented three machine learning (ML) models: Random Forest [37], XGBoost [38], and LightGBM [39].

These ML models were selected for their proven effectiveness in handling structured healthcare data and capturing complex, nonlinear relationships among predictors. Random Forest is a robust ensemble method known for reducing overfitting and accommodating a large number of features with minimal preprocessing [40]. XGBoost, or Extreme Gradient Boosting, is a highly efficient and scalable implementation of gradient boosting that has consistently outperformed traditional models in tabular data competitions and applied health research [38]. LightGBM, developed by Microsoft, offers faster training speed and lower memory usage compared to XGBoost, making it well-suited for large-scale datasets with high-dimensional features [41].

We trained and evaluated four models: logistic regression (as the baseline), Random Forest, XGBoost, and LightGBM. A key challenge in developing high-performing ML models lies in identifying the optimal set of hyperparameters. To address this, we employed GridSearchCV, an exhaustive grid search technique with cross-validation, to systematically tune hyperparameters and prevent overfitting.

For Random Forest, the grid search explores 48 different parameter combinations, examining n_estimators (100, 200), max_depth (10, 15, None), min_samples_split (2, 5), min_samples_leaf (1, 2), and max_features (‘sqrt’, ‘log2’). The XGBoost grid contains 32 combinations focusing on n_estimators (100, 200), max_depth (3, 6), learning_rate (0.1, 0.2), subsample (0.8, 0.9), and colsample_bytree (0.8, 0.9). LightGBM has the most extensive grid with 64 combinations, adding num_leaves (31, 50) to the XGBoost parameters. This systematic exploration ensures thorough coverage of the hyperparameter space while maintaining computational feasibility.

The cross-validation strategy employs RepeatedStratifiedKFold with 3 folds and 2 repeats, resulting in 6 total evaluations per parameter combination. Stratification was particularly important because, under the reviewer-requested quartile-based definition of UTI_high, the dataset necessarily exhibits class imbalance (25.1% positive class in training and 18.6% in testing, roughly 3:1 and 4:1 ratios). Preserving this distribution within each fold was essential to prevent bias during hyperparameter tuning. In total, the grid search involved 864 model evaluations (144 parameter combinations across the three algorithms × 6 cross-validation iterations).

The tuning process identified optimal configurations and corresponding cross-validation performance: Random Forest achieved the best performance with a CV score of 0.7736 (n_estimators = 200, max_depth = None, max_features = ‘sqrt’, min_samples_leaf = 1, min_samples_split = 2). XGBoost followed with a CV score of 0.7098 (n_estimators = 200, max_depth = 6, learning_rate = 0.2, subsample = 0.9, colsample_bytree = 0.9), while LightGBM achieved a CV score of 0.7052 (n_estimators = 200, max_depth = 6, learning_rate = 0.2, subsample = 0.8, colsample_bytree = 0.8, num_leaves = 50). Together, these results demonstrate both the rigor of the tuning strategy and the relative effectiveness of Random Forest under the outcome structure.

Model performance was assessed using metrics appropriate for binary classification tasks, including accuracy, precision, recall, F1-score, and the area under the receiver operating characteristic curve (AUC-ROC). These metrics enabled a comprehensive comparison between traditional logistic regression and machine learning models, highlighting improvements in predictive power and model robustness.

Next, we used Shapley Additive exPlanations (SHAP) to interpret the best-performing machine learning model by visualizing feature importance and examining the relationship between key predictors and the target outcome. SHAP values provide a consistent and theoretically grounded approach to quantify each feature’s contribution to individual predictions [42]. This technique allowed us to not only identify the most influential variables associated with high UTI rates in skilled nursing facilities but also to understand the direction and magnitude of their effects.

## 3. Results

### 3.1. Sample Descriptives

Table 3 presents descriptive statistics for key numeric variables before and after imputation. The dataset includes up to 94,877 observations spanning the years 2019 to 2024. Overall, post-imputation distributions closely resembled the original data, with minimal shifts in central tendency and dispersion. The number of staffed beds and its imputed counterpart exhibited similar distributions, with means around 112 and standard deviations between 46 and 48, indicating moderate variability across facilities. The minimum number of staffed beds was 8, and the 75th percentile reached 136, suggesting that most facilities were small to mid-sized. Both the original and imputed alos displayed substantial variability (*M* = 160 days; *SD* = 97), with a wide range from 1 to over 380 days, reflecting considerable differences in patient stay durations. The UTI rate demonstrated a maximum value of 32.35 and a 75th percentile of 2.89, indicating that while most facilities had low UTI rates, a small subset experienced disproportionately high rates.

Table 4 displays the frequencies and percentages calculated for categorical variables across the dataset after processing. The data were evenly distributed across six years (2019–2024), with each year representing approximately 16.5–16.8% of the total sample. Geographic classification indicated that 69.04% of facilities were located in urban areas (coded as 0), while 30.96% were rural (coded as 1). Ownership status revealed that 71.83% of facilities operated as for-profit entities, and 10.17% were government-owned. Facility quality, as measured by CMS star ratings, showed a relatively balanced distribution, with the largest proportion of facilities rated 1 star (24.68%) and the smallest rated 5 stars (16.4%).

Table 5 summarizes the average UTI Rates by SNF-Year after processing, where the UTI_Rate is the Definitive value for the percentage of long-stay residents in a Skilled Nursing Facility who have experienced a UTI during a given reporting period, typically measured for those who have been in the facility for 101 days or more. There was a definite downward trend in the mean value from 2019 (*M* = 2.61) to 2024 (*M* = 1.87). However, the sample size variation was small, ranging from 13,851 in 2019 to 14,071 in 2024, indicating the trend was not likely to be the result of sample size variation.

### 3.2. Machine Learning Results

Table 6 presents the performance metrics of each model after hyperparameter tuning. The evaluation includes Accuracy, ROC-AUC, F1-Score, and Area Under the Precision-Recall Curve (AUC-PR), which together offer a comprehensive assessment of model performance on a binary classification task.

As shown in Table 6, Random Forest demonstrates the highest bootstrap ROC-AUC (0.914) and the best F1-Score (0.467) among all models, indicating superior performance on training data. However, when evaluating true generalization performance on test data, Random Forest shows good but not excellent performance with a test AUC of 0.778, compared to XGBoost (0.741), LightGBM (0.732), and Logistic Regression (0.661). The 17.2% gap between bootstrap AUC (0.914) and test AUC (0.778) indicates potential overfitting to training data patterns, a common issue with complex ensemble methods.

**Figure 1:**
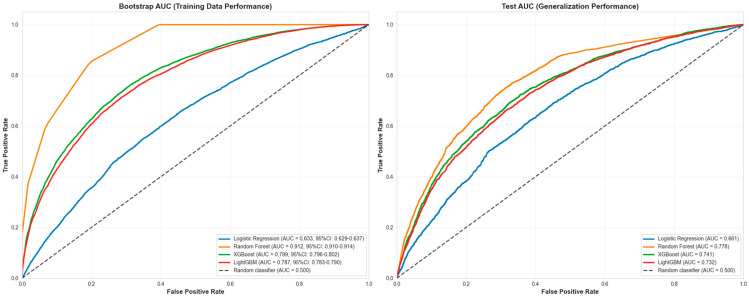
ROC-AUC Curve Comparison. **Left** panel shows ROC curves using training data predictions (bootstrap AUC), representing model performance on training data. **Right** panel shows ROC curves using test data predictions (test AUC), representing true generalization performance.

The side-by-side ROC curves comparison (Figure 1) illustrates this performance gap clearly. The left panel shows Random Forest’s bootstrap performance curve positioned very high, approaching the top-left corner (0,1) with AUC = 0.914, indicating excellent discrimination on training data. However, the right panel shows the test performance curve positioned lower with AUC = 0.778, representing the model’s true generalization ability. This visual comparison demonstrates that while Random Forest achieves the highest training performance, its test performance, while still competitive, is more modest and consistent with the other models’ test performance range.

**Figure 2:**
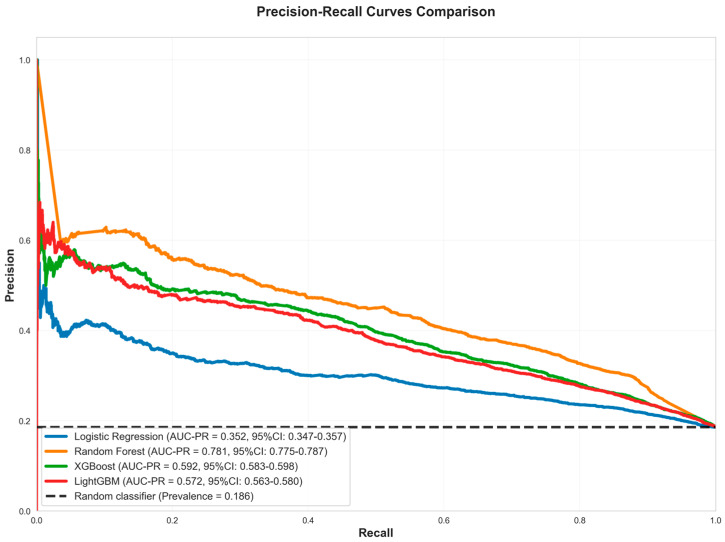
Precision-Recall Curve Comparison.

**Figure 3:**
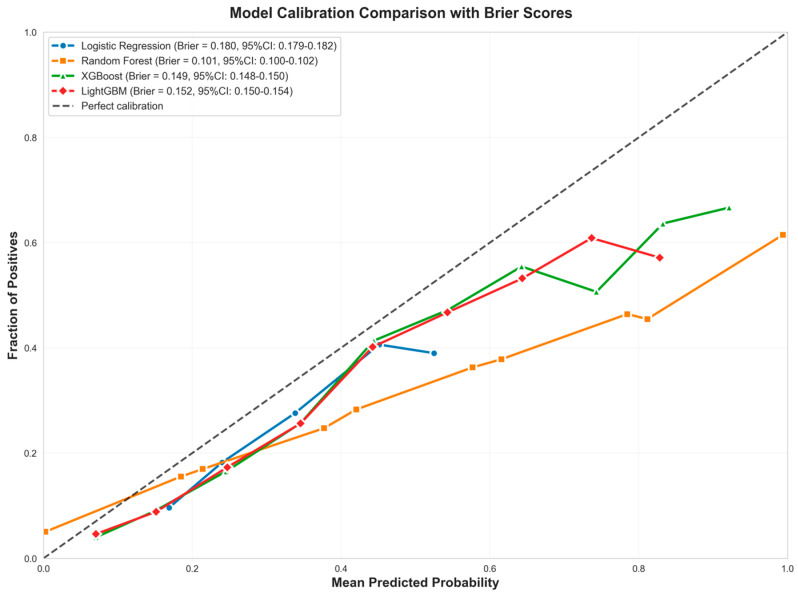
Calibration Curves Comparison.

Random Forest maintains competitive accuracy (0.794) and provides the strongest discrimination in the bootstrap ROC curves, though its calibration curve shows overconfidence at higher predicted probabilities. The performance gap between bootstrap and test AUC values highlights the importance of evaluating models on independent test data to assess true generalization performance, particularly when dealing with temporal data where patterns may shift between training and test periods. Figure 2 presents the Precision-Recall Curve Comparison. In Figure 3, Random Forest’s curve departs below the diagonal in mid-to-high probability bins, signaling overconfident estimates despite achieving the lowest Brier score (=0.101 with tight CI), which measures overall probabilistic accuracy; logistic regression tracks the diagonal more closely but with a higher Brier score, illustrating better visual calibration yet less accurate probabilities in aggregate. Taken together, these results indicate that Random Forest offers the best overall model for this task—top ROC-AUC (=0.912), highest AUC-PR, and the best F1—while requiring caution if absolute risks are used, where post hoc calibration (e.g., isotonic or Platt scaling) can improve probability reliability.

**Figure 4:**
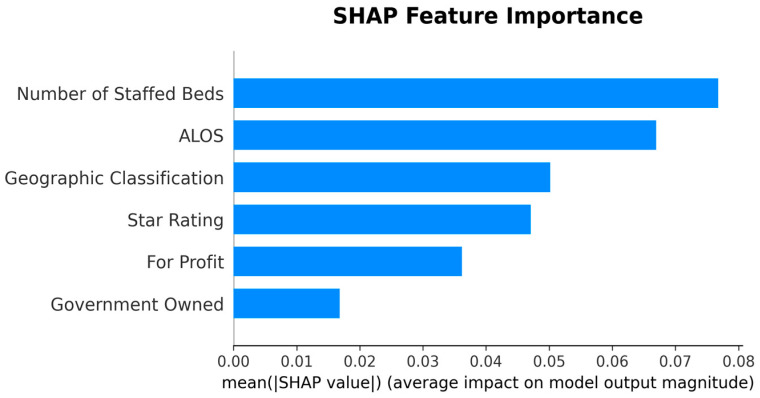
SHAP Feature Importance. Note: the SHAP Feature Importance reflects magnitude, not direction.

Given the study objective of identifying SNFs with high UTI risk, the Random Forest model was selected for downstream interpretation; SHAP analyses were then conducted to explain its predictions under this operating context. Table 7 presents the features ranked by their mean absolute SHAP values, which quantify the average magnitude of each feature’s contribution to the model’s output. Figure 4 visualizes these results, providing an overview of the relative importance of each feature.

Table 7 and Figure 4 indicate that facility characteristics, particularly number of staffed beds and ALOS, were the most influential predictors of high UTI rates, followed by Geographic Classification (rural vs. urban) and SNF’s star rating. However, it is important to note that SHAP values in this summary reflect only the magnitude of impact, not the direction. In other words, features with high mean SHAP values are important, but the summary does not indicate whether they increase or decrease the predicted risk.

**Figure 5:**
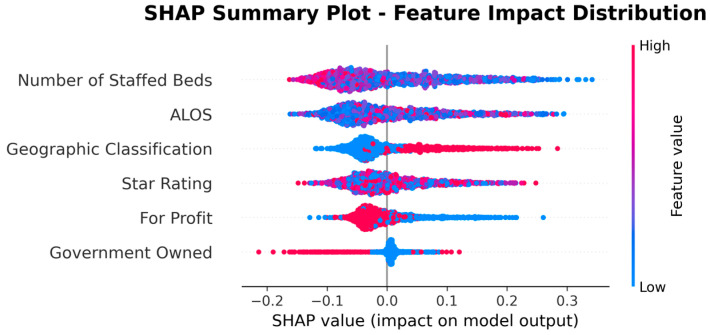
SHAP Summary (beeswarm) Plot.

To address this limitation, we present the SHAP Summary (beeswarm) plot (Figure 5), which ranks features by their average impact on model predictions, with Number of Staffed Beds as the most important predictor, followed by ALOS, Geographic Classification, Star Rating, For Profit, and Government Owned. While the summary plot effectively displays feature importance, its visual complexity can make directionality less clear, especially when high and low feature values overlap in their SHAP contributions.

**Figure 6:**
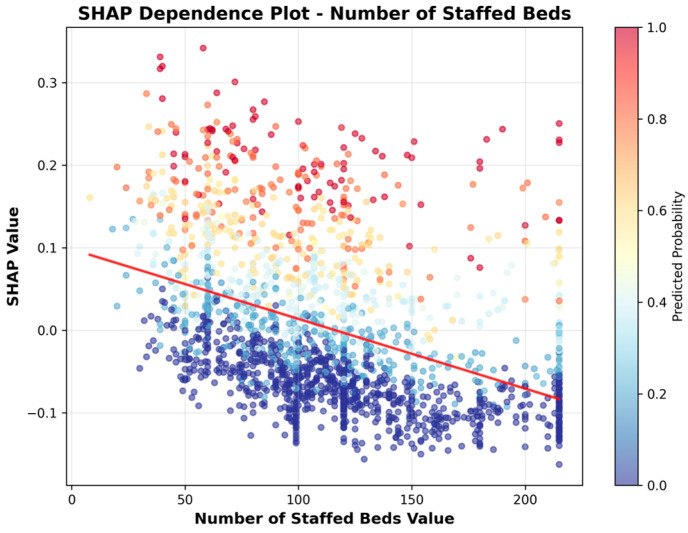
SHAP Dependence Plot for Number of Staffed Beds.

**Figure 7:**
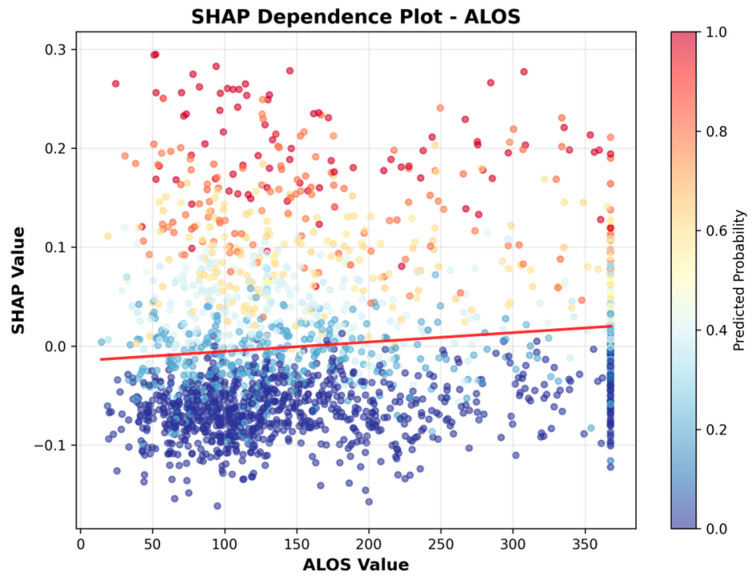
SHAP Dependence Plot for ALOS.

**Figure 8:**
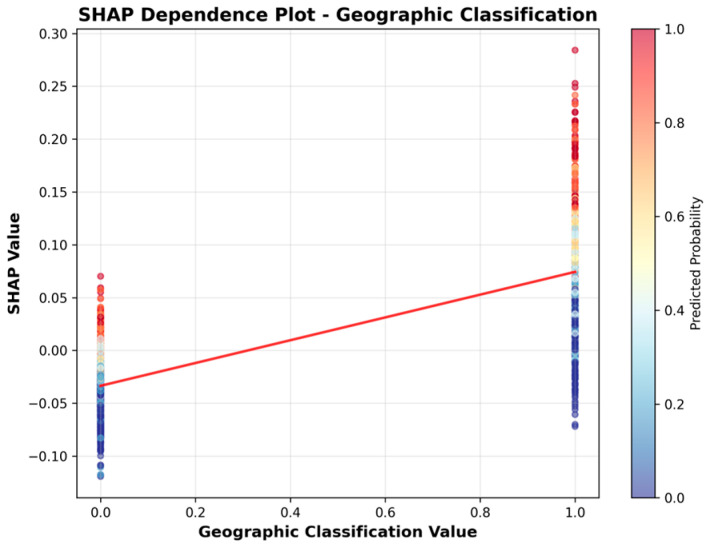
SHAP Dependence Plot for Geographic Classification.

**Figure 9:**
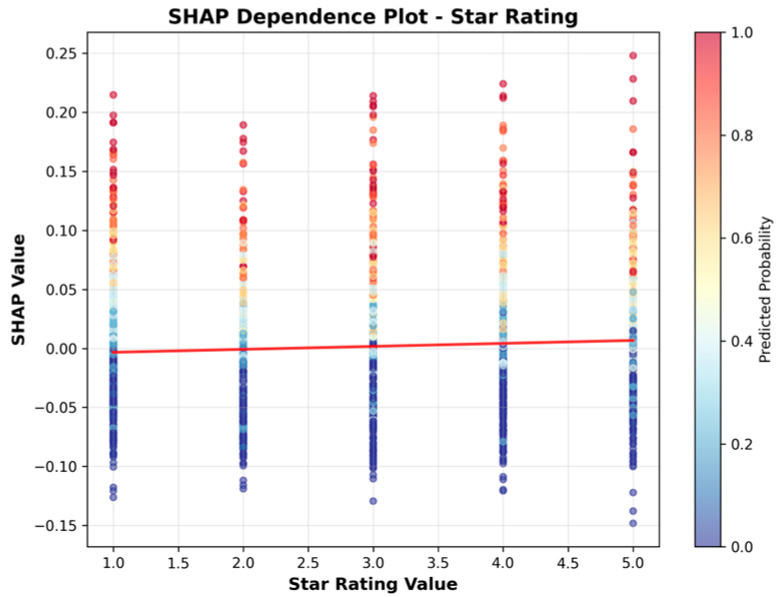
SHAP Dependence Plot for Star Rating.

**Figure 10:**
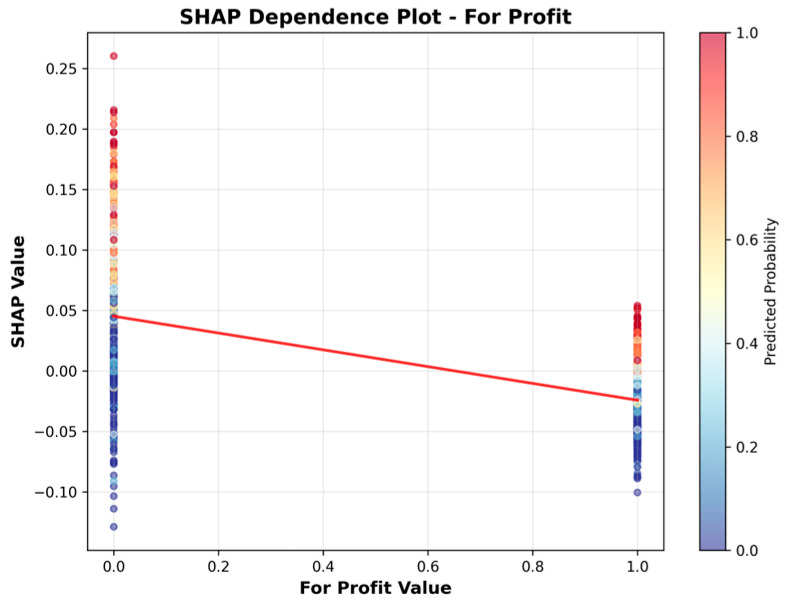
SHAP Dependence Plot for Profit.

**Figure 11:**
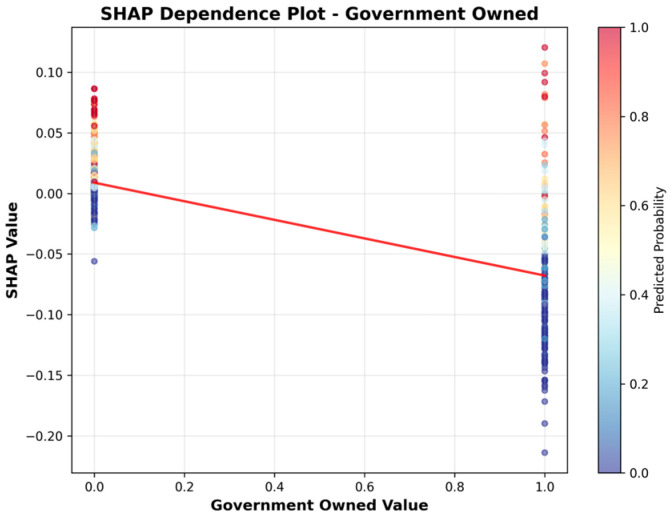
SHAP Dependence Plot for Government Owned.

To more clearly illustrate the direction of association, we include SHAP dependence plots (Figure 6, Figure 7, Figure 8, Figure 9, Figure 10 and Figure 11). For example, the dependence plot for Number of Staffed Beds shows a negative relationship: facilities with fewer staffed beds have higher SHAP values, indicating increased predicted UTI risk, while those with more staffed beds have lower risk. Similarly, the dependence plot for Geographic Classification demonstrates that rural status is associated with a higher predicted risk. These plots provide a direct visualization of how feature values influence the model’s output, clarifying both importance and direction.

Figure 6, Figure 7, Figure 8, Figure 9, Figure 10 and Figure 11 SHAP Dependence Plots for Features.

As shown in Figure 6, the SHAP dependence plot for Number of Staffed Beds reveals a clear negative association: facilities with fewer staffed beds have higher SHAP values, indicating increased predicted risk for high UTI rates. Conversely, facilities with more staffed beds generally receive lower SHAP values, reflecting reduced predicted risk. This pattern suggests that smaller facilities are more likely to be identified as high-risk for UTIs by the model.

As shown in Figure 7, facilities with longer average lengths of stay (ALOS) generally have slightly higher SHAP values, indicating an association between higher ALOS and increased predicted UTI risk.

Figure 8 demonstrates that rural status (Geographic Classification = 1) is linked to higher SHAP values, meaning rural facilities are predicted to have a higher risk of UTIs than urban ones.

Figure 9 shows a weak positive association, where facilities with higher star ratings tend to have slightly higher SHAP values and, therefore, modestly greater predicted UTI risk.

Figure 10 reveals that for-profit status (value = 1) is also linked to lower SHAP values, suggesting that for-profit facilities generally show a lower predicted risk of UTIs compared to nonprofit facilities.

In Figure 11, being government-owned (value = 1) is associated with lower SHAP values, indicating that government-owned facilities are predicted to have a lower risk of UTIs compared to non-government-owned ones.

## 4. Sensitivity Analyses

To maintain conciseness and focus on the optimal model, we report sensitivity analyses using only the Random Forest model, which achieved the best performance in our main analysis (ROC-AUC = 0.912). While we evaluated all four models (Random Forest, XGBoost, LightGBM, Logistic Regression) in our sensitivity analyses, we present results for Random Forest only to avoid redundant reporting and maintain manuscript clarity.

### 4.1. Geographic Classification Sensitivity

To address concerns that geographic location might dominate model predictions, we conducted a sensitivity analysis by removing the geographic classification feature (which ranked third in importance in our main analysis) and analyzing rural and urban facilities separately. We re-analyzed our data using only the five modifiable facility characteristics (number of staffed beds, average length of stay, for-profit status, government ownership, and star rating) within rural and urban facility subgroups.

The performance difference between rural and urban facilities was 0.034 ROC-AUC points, and the overall impact of removing geographic classification was 0.009 ROC-AUC points compared to the main analysis. These results suggest that geographic classification has minimal impact on model performance and that there are minimal differences in predictive performance across rural and urban facility settings. This sensitivity analysis demonstrates that our model’s performance is robust across different geographic settings, with modifiable facility characteristics showing consistent predictive value regardless of geographic location; see Table 8.

### 4.2. Grouped Facility Split Sensitivity Analysis

Table 9 presents the Sensitivity Analysis. To address potential data leakage from the same facilities appearing in both training and test periods, we conducted a sensitivity analysis using grouped facility and temporal splits. We randomly assigned 70% of facilities to the training set and 30% to the test set, ensuring no facility appeared in both sets. The performance difference between temporal validation (ROC-AUC = 0.912) and grouped facility split (ROC-AUC = 0.872) was 0.040 ROC-AUC points. This suggests minimal data leakage, with the temporal validation approach accurately reflecting model performance due to robust generalization. These results demonstrate that our model is robust against data leakage from facility-specific patterns and that the temporal validation approach does not overestimate performance due to overlapping facilities across time periods.

Interpretation and Clinical Relevance: The 99.7% facility overlap between training and test periods in our main analysis creates minimal data leakage, which does not significantly affect model performance estimates. Importantly, the choice between temporal and grouped facility splits addresses fundamentally different research questions. Our temporal split approach answers the clinically relevant question: “Can we predict UTI risk for our existing facilities next year?” This is particularly valuable for healthcare administrators who need to identify high-risk facilities within their current network for targeted interventions. In contrast, the grouped facility split addresses: “Can we predict UTI risk for completely new facilities?”

Given that skilled nursing facilities maintain consistent operational characteristics over time and healthcare administrators typically work with existing facility networks, the temporal split approach is more clinically relevant for our study objectives. Our results demonstrate that modifiable facility characteristics (staffing, length of stay, ownership, star rating) provide robust predictive value even when accounting for potential facility-specific patterns. Future studies focusing on predicting performance for entirely new facilities entering the market may benefit from facility-level split approaches, but our temporal validation provides appropriate performance estimates for the clinical decision-making context addressed in this study.

## 5. Discussion

This demonstrates that a machine learning model utilizing SNF characteristics can outperform traditional logistic regression in predicting skilled nursing facilities at high risk for HAIs, based on facility characteristics, which supports our primary hypothesis. In support of our second hypothesis, our findings indicate that facility-level characteristics are influential predictors of UTIs. The top four predictors were the number of staffed beds, average length of stay, geographic location, and star rating. The fifth most influential factor was the facility’s for-profit status.

### 5.1. Comparison to Previous Research

This study introduces a novel research perspective by focusing on facility-level risk prediction for UTIs, diverging from prior work that has predominantly examined patient-level risk factors. Earlier studies have concentrated on individual characteristics, such as age, gender, comorbidities, procedures, and treatments, in relation to specific infections like surgical site infections [26], hospital-acquired urinary tract infections [9], and healthcare-associated pneumonia [27]. Our approach leverages facility-level variables, including staffing levels, ownership type, and geographic location, to predict HAI risk, in contrast with earlier studies focused on acute care hospitals or intensive care units. While one prior study did examine hospital characteristics such as patient safety climate, adherence to standard precautions, nurse staffing, and ownership, it did not incorporate geographic factors (e.g., rural vs. urban), number of staffed beds, or aim to predict HAI risk at the facility level [10].

The value of predictive modeling in infection control is further supported by Zhao et al. (2023), who used machine learning to forecast UTIs in neurocritical care patients following intracerebral hemorrhage [8]. Their model achieved strong predictive performance and highlighted the importance of dynamic, context-specific variables—an approach we extend to the facility level in SNFs. Similarly, Liu et al. (2024) applied decision tree analysis to identify catheter-associated UTI risk factors in neurosurgical ICU patients, emphasizing clinical variables such as catheter duration, diabetes, and post-surgical status [6]. While both studies demonstrate the effectiveness of patient-level predictive modeling in acute care settings, our study shifts the focus to structural and organizational factors in post-acute SNFs. This broader lens enables identification of systemic vulnerabilities and informs facility-level interventions, such as staffing policy adjustments and standardized diagnostic protocols, to reduce UTI incidence across diverse care environments.

Additionally, by emphasizing facility-level predictors, this study enhances the interpretability of the predictive model and identifies actionable areas for policy and clinical intervention. These insights can inform targeted strategies to reduce urinary tract infection rates in SNFs, guide resource allocation, and support regulatory oversight. For example, staffing policy changes to increase nurse practitioner coverage, investments in nurse-led hygiene education, and proactive monitoring protocols may help reduce UTI incidence [43]. Moreover, SNFs with longer average lengths of stay may benefit from standardized diagnostic criteria to mitigate the risk of drug-resistant infections [44].

### 5.2. SNF Facility Characteristics

Our model predictors were the SNF’s characteristics. We examined SNFs from 2019 to 2024, revealing considerable variation in facility characteristics and patient outcomes. A key finding was a steady decline in the average UTI rate from 2019 (*M* = 2.61) to 2024 (*M* = 1.87). Because the number of facilities studied each year showed little variation, this downward trend likely reflects real improvements in infection control practices rather than variations in sample size.

The sample was predominantly urban, for-profit, and non-governmental facilities. These star quality ratings showed a relatively balanced distribution, with the largest proportion of facilities rated 1 or 2 stars, indicating that many SNFs may be operating below optimal quality standards.

Facility size varied greatly, with the number of staffed beds ranging from 8 to 223 beds (*M* = 112), indicating a mix of small community-based facilities and large institutional providers. Similarly, the ALOS varied significantly, from 1 to 382 days, highlighting the diverse patient populations and care needs across SNFs.

### 5.3. Study Results and Hypotheses

The study results mostly aligned with expectations. For instance, the number of staffed beds had a negative association with lower SHAP values, suggesting that smaller facilities tend to have a higher predicted risk of UTIs.

As expected, facilities with a longer ALOS had a higher predicted UTI risk. Moreover, rural facilities were predicted to have a higher UTI risk compared to urban facilities, which may be explained by staffing issues, budget constraints, and lack of specialists on staff at rural facilities, although further investigation is needed.

Star rating had a weak positive association with higher star ratings moderately associated with greater UTI risk. Some features, such as for-profits and government-owned facility status, had minimal impact on the model’s predictive performance.

### 5.4. Limitations

The main limitation was the variation in facility size, staffing models, ownership types, and patient populations across SNFs, which may produce confounding variables that this study did not consider. For example, diverse patient populations and care needs across SNFs may lead to differences in case mix, staffing models, and resource allocations that were not considered. Additionally, future studies incorporating resident-level clinical data, like case mix acuity, could provide valuable insights into how case mix affects UTI prediction models. Thus, a data set with less facility characteristic variation may have more significant results. There are also limitations due to potential temporal leakage due to a random split and the proposed solution (time-based validation), justification of the outcome threshold, sensitivity analyses, and lack of external validation.

Another limitation is the variability in the quality of self-reported data. Inconsistencies in reporting STAR quality ratings and UTI outcomes across facilities may affect the accuracy of model predictions.

Geographical distribution presents a limitation. The sample was predominantly composed of urban, for-profit, and non-governmental facilities, which may limit the generalization of results to rural, nonprofit, government-owned SNFs. Furthermore, it should be noted that while SHAP values indicate associations and enhance ML interpretability, they do not imply causation.

## 6. Conclusions

This study demonstrates the value of using facility-level characteristics to predict the risk of UTIs in SNFs through machine learning models. Our findings indicate that models utilizing factors such as rural location, number of staffed beds, ownership type, and ALOS can outperform traditional logistic regression in identifying high-risk facilities. This study’s findings are predictive rather than causal and do not establish direct cause–effect relationships. To enhance practical utility, future research should consider external validation or pilot testing of a facility-level alert tool that flags high-risk SNFs based on predictive indicators. Results from this study can inform infection prevention efforts in post-acute care settings.

## Figures and Tables

**Table 1:** Percent Missingness by Variable by Year (*N* = 94,877).

Statistic	2019	2020	2021	2022	2023	2024	Imputation Strategy
accreditation	99.69	99.69	99.68	99.68	99.68	99.69	Dropped
aco_affiliations	95.47	95.48	95.47	95.49	95.49	95.47	Dropped
alos	3.65	3.64	3.64	3.64	3.68	3.75	Median
for_profit	2.87	2.85	2.84	2.84	2.86	2.94	Mode
geographic_classification	0	0	0	0	0	0	Not imputed
government_owned	2.87	2.85	2.84	2.84	2.86	2.94	Mode
num_staffed_beds	3.51	3.5	3.51	3.5	3.55	3.62	Median
nurse_hrs	100	100	100	100	100	0.78	Dropped
pt_hrs	100	100	100	100	100	0.78	Dropped
star_rating	0.69	0.7	0.72	0.72	0.76	0.85	Mode
uti_rate	0	0	0	0	0	0	Not imputed
year	0	0	0	0	0	0	Not imputed

**Table 2:** VIF by numeric variables.

Feature	VIF
For Profit	1.48
Government Owned	1.44
Geographic Classification	1.13
Num Staffed Beds	1.1
ALOS	1.07
Star rating	1.06

**Table 3:** Sample Numeric Factor Descriptive Statistics for SNF-Year (*N* = 94,877).

Statistic	num_staffed_beds	alos	uti_rate *	alos_imputed	num_staffed_beds_imputed
count	91,471	91,345	94,877	94,877	94,877
mean	112.62	160.12	2.02	157.85	112
std	47.72	97.6	2.48	93.37	45.9
min	8	1	0	1	8
25%	78	88.51	0.06	89.71	80
50%	107	129.53	1.25	129.53	107
75%	136	205.96	2.89	200.92	134
max	223	382.14	32.35	367.74	215

* Note: uti_rate is coded 0/1.

**Table 4:** Sample Categorical Factor Frequencies for SNF-Year (*N* = 94,877).

Variable	Category	*n*	Percentage (%)
year	2019	13,851	16.51
	2020	13,917	16.59
	2021	13,967	16.65
	2022	14,021	16.71
	2023	14,068	16.77
	2024	14,071	16.77
geographic_classification	0	65,503	69.04
	1	29,374	30.96
for_profit	0	26,726	28.17
	1	68,151	71.83
government_owned	0	85,225	89.83
	1	9652	10.17
star_rating	1	23,411	24.68
	2	20,403	21.50
	3	19,002	20.03
	4	16,502	17.39
	5	15,559	16.4
for_profit	0	26,726	28.17
	1	68,151	71.83

**Table 5:** Average UTI Rates by SNF-Year (*N* = 94,877).

Year	*n* *	Mean
2019	13,851	2.61
2020	13,917	2.47
2021	13,967	2.35
2022	14,021	2.29
2023	14,068	2.11
2024	14,071	1.87

* Notes: One SNF-year represents one facility observed over one year; total sample size represents the number of facility-years included in the unprocessed data, meaning some facilities may contribute data across multiple years.

**Table 6:** Model Performance Summary.

Model	Accuracy	ROC-AUC *	F1-Score	AUC-PR
Random Forest	0.794	0.914	0.467	0.438
XGBoost	0.814	0.8	0.253	0.392
LightGBM	0.814	0.789	0.238	0.383
Logistic Regression	0.81	0.634	0.07	0.298

* ROC-AUC values represent bootstrap sampling performance on training data (95% CI provided in text).

**Table 7:** Mean Absolute SHAP Value of Features in the Fine-tuned Random Forest Model.

Rank	Feature	|SHAP| Mean
1	Number of Staffed Beds	0.0768
2	ALOS	0.0669
3	Geographic Classification	0.0502
4	Star Rating	0.0471
8	For Profit	0.0362
9	Government Owned	0.0168

Note: the SHAP summary reflects magnitude, not direction.

**Table 8:** Rural and Urban Sensitivity Analysis.

Analysis *	ROC-AUC (95% CI)	F1-Score (95% CI)	Test Sample Size
Main Analysis	0.912 (0.910–0.914)	0.662 (0.656–0.668)	14,071
Rural	0.920 (0.918–0.921)	0.637 (0.631–0.644)	9746
Urban	0.886 (0.882–0.891)	0.694 (0.686–0.703)	4325

* Note: Main Analysis (all 6 features, Rural and Urban (5 features, no geographic).

**Table 9:** Split Method Temporal and Grouped Facility.

Split Method	ROC-AUC (95% CI)	F1-Score (95% CI)	Train Facilities	Test Facilities	Test Samples
Temporal Split (Original)	0.912 (0.910–0.914)	0.662 (0.656–0.668)	14,127	14,071	14,071
Grouped Facility Split	0.872 (0.869–0.874)	0.576 (0.569–0.582)	9915	4250	28,373

## Data Availability

Publicly available compiled datasets were accessed with the assistance of the Definitive Healthcare website (found here: https://www.defhc.com/, accessed on 20 April 2025). This is a subscription-based resource. As such, data are proprietary and cannot be publicly reposted, redistributed, or shared.

## Abbreviations

The following abbreviations are used in this manuscript:

ACO	Accountable Care Organization
ALOS	Average Length of Stay
AUC-ROC	Area Under The Receiver Operating Characteristic Curve
CMS	Centers for Medicare & Medicaid Services
HAI	Healthcare-Associated Infection
IQR	Interquartile Range
ML	Machine Learning
ROC-AUC	Receiver Operating Characteristic–Area Under the Curve
ROC	Receiver Operating Characteristic
SHAP	Shapley Additive Explanations
SNF	Skilled Nursing Facility
UTI	Urinary Tract Infection
VIF	Variance Inflation Factor
